# Supplementary figures and images for: The Neuro-Protective Effects of the TSPO Ligands CB86 and CB204 on 6-OHDA-Induced PC12 Cell Death as an In Vitro Model for Parkinson’s Disease
Source: Biology (Basel). 2021 Nov 15;10(11):1183. doi: 10.3390/biology10111183 (PMC8615274; doi:10.3390/biology10111183)

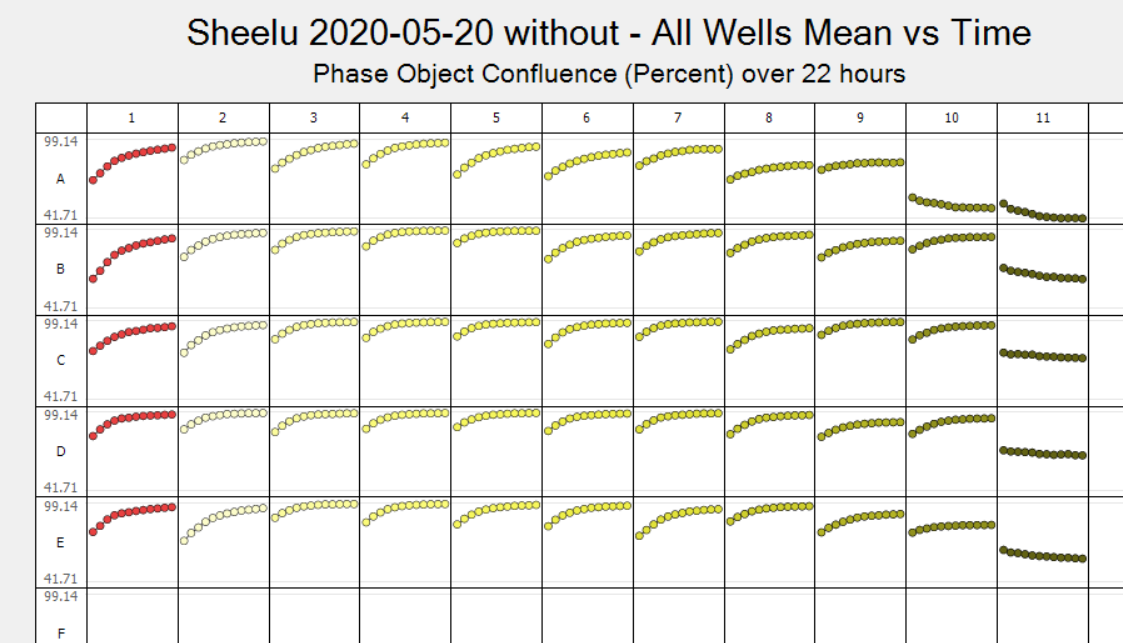

Supplement: Supplementary file 1 [file biology-10-01183-s001.zip › 6-OHDA dose response.png]

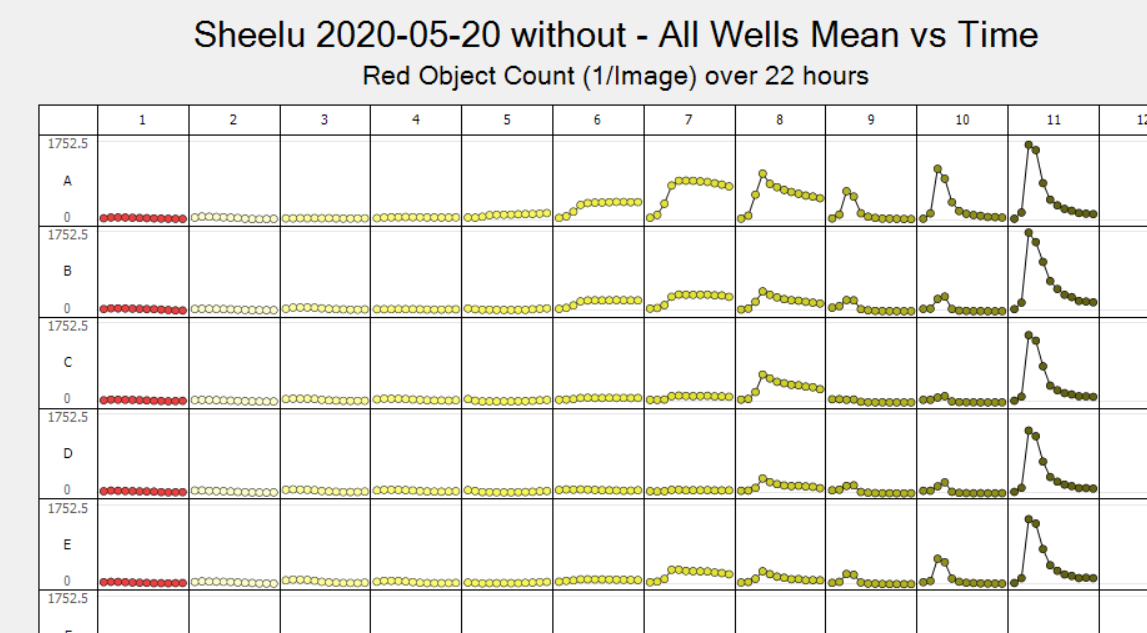

Supplement: Supplementary file 1 [file biology-10-01183-s001.zip › 6-OHDA- dose response 1.png]
